# Weekly administration of rapamycin improves survival and biomarkers in obese male mice on high-fat diet

**DOI:** 10.1111/acel.12211

**Published:** 2014-03-22

**Authors:** Olga V Leontieva, Geraldine M Paszkiewicz, Mikhail V Blagosklonny

**Affiliations:** Cell Stress Biology, Roswell Park Cancer InstituteBuffalo, NY, 14263, USA

**Keywords:** aging, anti-aging agent, longevity, mammalian or mechanistic target of rapamycin, mortality, obesity, rapamycin

## Abstract

Recent discoveries have revealed the key role of mTOR (target of rapamycin) in aging. Furthermore, rapamycin extends lifespan in mice, especially in female mice. Here, we treated obese male mice on high-fat diet with rapamycin given intermittently: either weekly (once a week) or alternating bi-weekly (three injections every other week). While only marginally reducing obesity, intermittent administration of rapamycin significantly extended lifespan. Significance was achieved for weekly treated group and for the three rapamycin-received groups combined. In weekly treatment group, 100% mice were alive by the age of 2 years, whereas 60% of mice died in untreated group by this age. The effect of weekly treatment on survival was highly significant and cannot be fully explained by partial reduction in obesity. Alternating bi-weekly treatments seem to be less effective than weekly treatment, although effects of additional factors (see Discussion) may not be excluded. After one year of treatment, all survived mice were sacrificed 8 days after the last administration of rapamycin to avoid its direct interference with parameters examined. Fasting levels of cardiac and hepatic p-S6, a marker of mTORC1 activity, were lower in weekly treatment group compared with control mice. In contrast, levels of p-Akt (S473), glucose, triglycerides and insulin were unchanged, whereas leptin and IGF-1 tended to be lower. Thus, weekly treatment with rapamycin may slow down aging in obese male mice on high-fat diet.

## Introduction

mTOR (mammalian or mechanistic target of rapamycin) is involved in organismal aging (Blagosklonny & Hall, 2009; Stanfel *et al*., 2009; Kapahi *et al*., 2010; Sengupta *et al*., 2010b; Bjedov & Partridge, 2011; Zoncu *et al*., 2011; Cornu *et al*., 2012; Flynn *et al*., 2013; Johnson *et al*., 2013). Inhibition of mTOR suppresses geroconversion from reversible arrest to senescence (Demidenko *et al*., 2010; Leontieva *et al*., 2012c, 2013a). An increasing number of studies have demonstrated that rapamycin extends lifespan in mice and prevents age-related pathologies including cancer (Harrison *et al*., 2009; Miller *et al*., 2011; Anisimov *et al*., 2011b, 2010; Ramos *et al*., 2014; Wilkinson *et al*., 2012; Spong & Bartke, 2012; Selman & Partridge, 2012; Neff *et al*., 2013; Ye *et al*., 2013; Flynn *et al*., 2013; Zhang *et al*., 2014). Given that rapamycin is a clinically approved drug, it potentially can be used to slow aging in humans. The effect of rapamycin on longevity has been studied in mice on regular diet. Yet, most humans abuse high-calorie diet and suffer from obesity. Will obesity and high-fat (HF) diet counteract beneficial effects of rapamycin? Also, rapamycin extends lifespan in male mice to a lesser degree compared with female mice (Harrison *et al*., 2009; Miller *et al*., 2011), probably in part due to different sensitivity to rapamycin (Leontieva *et al*., 2012a). Most previous studies used chronic (everyday) administration of rapamycin. Here, we investigated the effect of intermittent treatment with rapamycin followed by treatment-free breaks. Albeit based on theoretic considerations (Blagosklonny, 2012b), the choice of treatment schedules was relatively arbitrary. Two groups (R1 and R3) of mice on high-fat diet received 3 i.p. injections of 1.5 mg kg^−1^ or 0.5 mg kg^−1^ of rapamycin during one week followed by a treatment-free week (alternating bi-weekly schedule). Also we included a simple, every-week treatment with one injection of 1.5 mg kg^−1^ of rapamycin (weekly or R2 group). This weekly schedule (group R2) resulted in superior effects.

## Results

### Rapamycin tended to decrease body weight on HF diet

**Figure 1:**
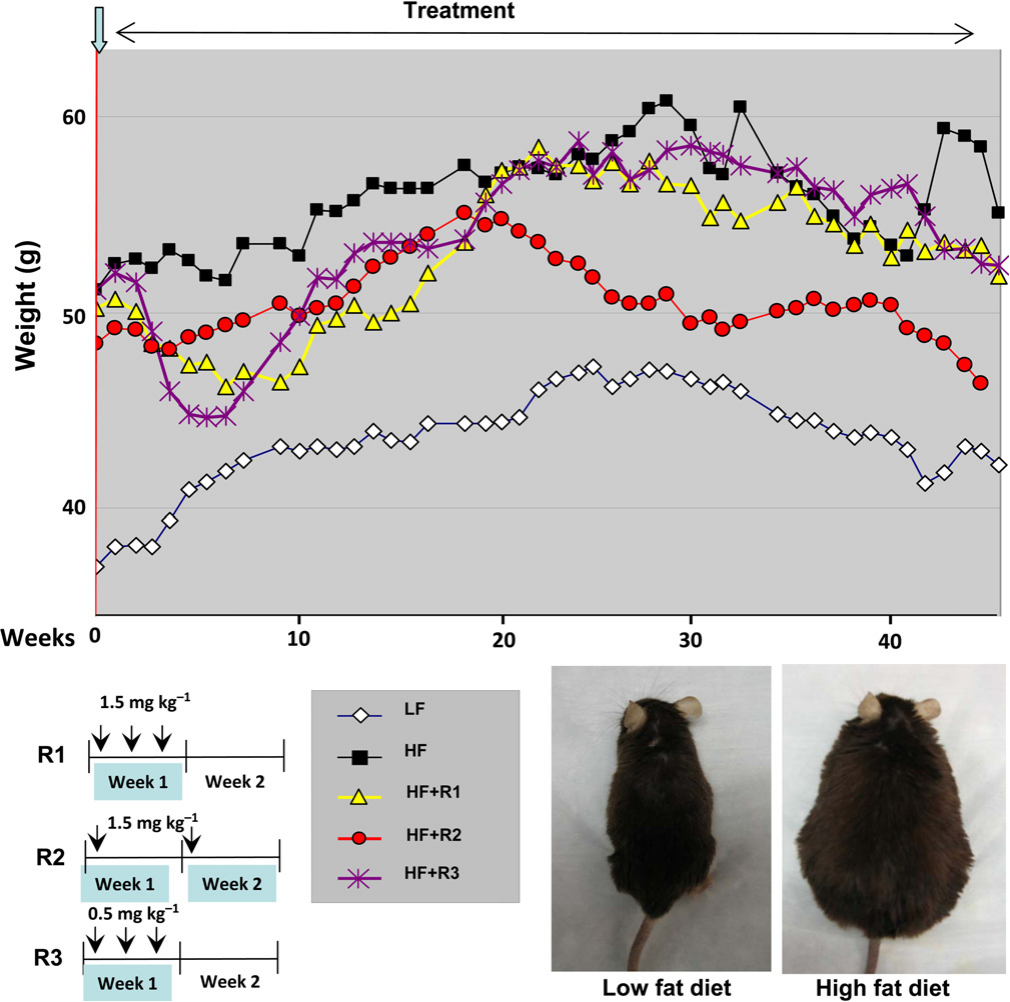
Weekly body weight during the experiment. Absolute body weights. Nine-month-old male mice were divided into five groups: low-fat (LF) diet control mice and four high-fat (HF) diet groups: untreated control (HF), R1 – 1.5 mg kg^−1^ rapamycin three times/week every other week; R2 group – 1.5 mg kg^−1^ per a week every week and R3 group – 0.5 mg kg^−1^ three times/week every other week. Treatment was continued for 11 months, and weight was measured every week. Upper panel – average weights for each group are presented. Bottom panel – schemas of rapamycin treatment schedules and photos of representative mice from low-fat and high-fat control groups are shown.

Three groups of male mice on high-fat diet were treated by i.p. injections with the following intermittent schedules: R1 – 1.5 mg kg^−1^ three times/week every other week; R2 (weekly schedule) – 1.5 mg kg^−1^ week^−1^; and R3 – 0.5 mg kg^−1^ three times per week every other week (Fig. 1). These groups of mice were fed with HF food for 3 months before the start of the current study (Experimental procedures, prehistory). So at the start of the treatment, all three treatment groups were obese (Fig. 1) and were similar to control HF diet group (compare weights at week 0 in Fig. 1). When this treatment started (Fig. 1), there was a transient decrease in absolute body weight in three rapamycin-treated groups (Fig. 1). In contrast, control mice on high-fat diet continued to gain weight until reaching a plateau after 8 months (Fig. 1 and Fig. S1, Supporting information). By the end of the experiment, mice in group R2 showed tendency to weigh less than control HF mice (Fig. 1). There was a statistically significant, albeit transient reduction in weight gain in R2 group compared with HF control group (Fig. S1, Supporting information indicated by asterisks).

### Rapamycin extends lifespan in obese male mice

**Figure 2:**
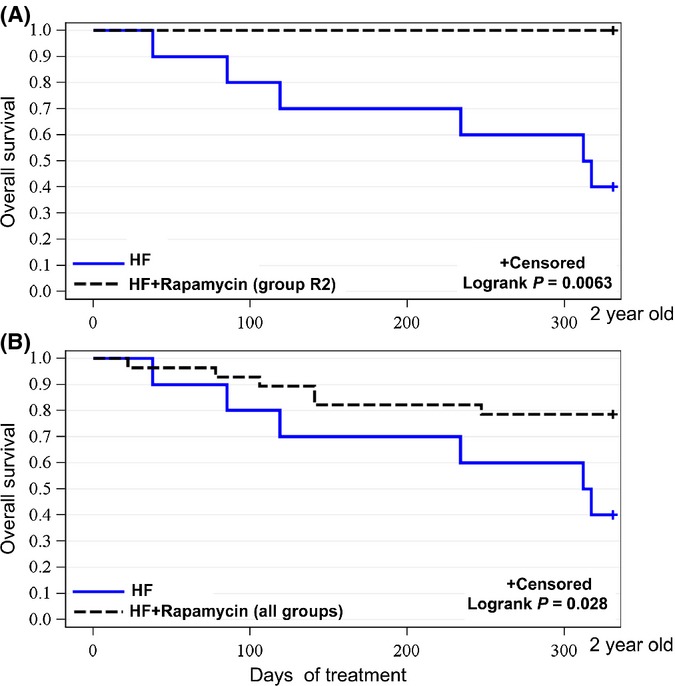
Rapamycin extended lifespan of mice on high-fat (HF) diet. (A). Kaplan–Meier survival rate curve for control mice on HF diet (HF; *n* = 10) and mice treated with rapamycin following schedule R2 (HF+rapamycin (group R2); *n* = 9). (B). Kaplan–Meier survival rate curve for control mice on HF diet (HF; *n* = 10) and all groups of mice treated with rapamycin (all groups) taken together (*n* = 28).

Analysis of overall survival of individual rapamycin-treated groups versus HF control mice revealed that weekly treatment with rapamycin (schedule R2) significantly prevented morbidity and death in obese male mice on HF diet (Fig. 2A). Whereas 60% of control mice on high-fat diet died or were sacrificed due to morbidity, all mice in R2 group survived. (The cause of death was not always determined so we do not provide these data). There was a high statistically significant difference (*P* = 0.0063) in overall survival of mice in group R2 compared with control HF group (Fig. 2A). Survival rate of all three rapamycin-treated groups taken together for analysis also was significantly higher when compared to control HF group (*P* = 0.028, Fig. 2B). Thus, significance was achieved for group R2 and for the three rapamycin-received groups combined, relative to the HF group. Alternating bi-weekly treatments (groups R1 and R3) showed tendency to increase survival, but it was not statistically significant (Fig. S2, Supporting information).

### Levels of p-S6 in surviving mice

After eleven months of treatment, when 60% of mice in HF control group died, the experiment was stopped to examine the surviving mice. In all three rapamycin-treated groups, mice were sacrificed 8 days after the last treatment, to eliminate direct effects of rapamycin on examined parameters**.** We focused the study on the heart and the liver. Previous reports point to the heart (the cardiac muscle) as to also being very important tissue to measure phospho-S6 (Hua *et al*., 2011; Leontieva *et al*., 2012a, 2013b; Ramos *et al*., 2012; Flynn *et al*., 2013; Wu *et al*., 2013; Zhou *et al*., 2013). The heart is one of the most commonly studied organs in aging, and rapamycin treatment improves the heart condition. Furthermore, cardiovascular diseases are the most common cause of death in humans. Importantly, our previous studies implicate the cardiac phospho-S6 as a potential marker of longevity and animal hypertrophy (Leontieva *et al*., 2013b) (Leontieva *et al*., 2012a; Flynn *et al*., 2013).

**Figure 3:**
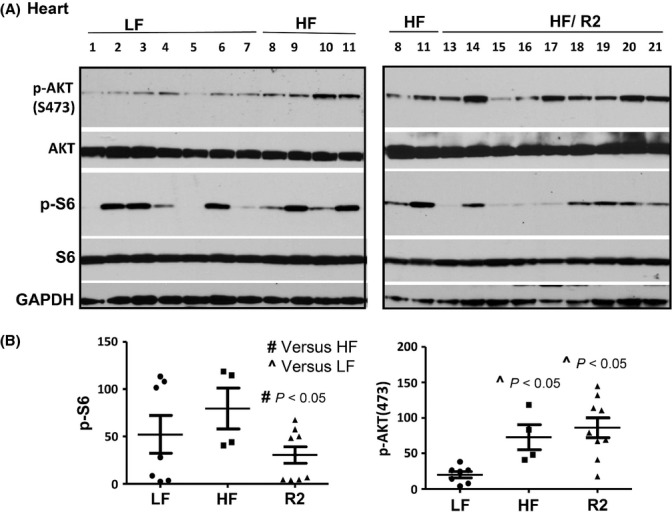
p-S6 in the heart of the survived mice. (A). Immunoblot analysis of protein lysates from the heart of mice on low-fat (LF) or high-fat (HF) diet: control (HF – untreated) or rapamycin treated (group R2 presented – HF/R2). Immunoblotting was performed with the indicated antibodies. Numbers indicate individual mice. (B). Quantitative analysis of data shown in Fig. 3A. Quantified intensities of p-S6 signal (left panel) and signal of p-AKT(Ser473) (right panel) presented as mean ± SE.

In R2 group, levels of cardiac p-S6 were statistically lower than in control HF group (Fig. 3). P-S6 reflects the activity of mTORC1, which is known to be involved in aging. We also measured phosphorylation of AKT at S473, which is in part a marker of mTORC2 activity. In contrast to p-S6, p-AKT(S473) was not decreased in R2 group (Fig. 3).

**Figure 4:**
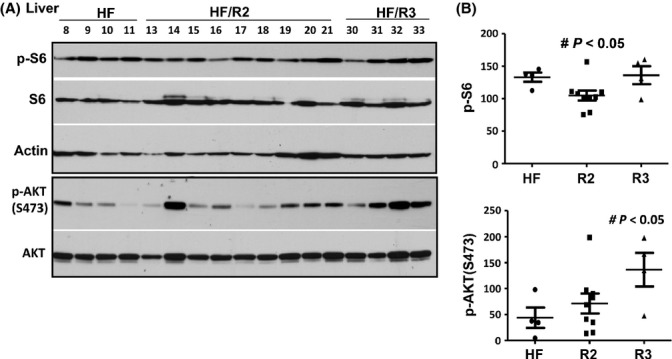
p-S6 and p-Akt in the liver of survived mice. (A). Immunoblot analysis of protein lysates from the liver of ~ 2-year-old male mice on high-fat (HF) diet: control (HF – untreated) or rapamycin treated (groups R2 and R3 presented – HF/R2, HF/R3). Numbers indicate individual mice. Equal loading was also confirmed by staining the membrane with Commassie Blue (Fig. S3, Supporting information). (B). Quantitative analysis of data shown in Fig. 4A. Quantified intensities of p-S6 signal and signal of p-AKT(Ser473) presented as mean ± SE.

We also evaluated these parameters in the liver. In agreement, levels of hepatic p-S6 were significantly lower in R2 group compared with HF control. Similar to the hearts, the hepatic levels of p-AKT(S473) had tendency to be higher in R2 group although it did not reach statistical difference in this particular test (Fig. 4). Noteworthy, the levels of p-S6 and p-Akt in the kidneys were not different in control and rapamycin-treated groups and p-S6 and p-Akt highly correlated (Fig. S4, Supporting information).

### Weekly treated mice did not have metabolic abnormalities

**Figure 5:**
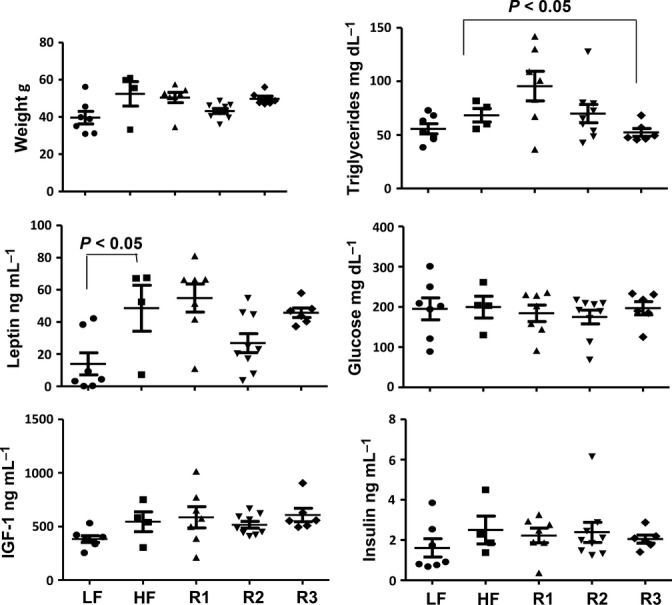
Metabolic parameters in survived mice. Weights and metabolic parameters (leptin, IGF1, triglycerides, glucose and insulin) were determined in fasted plasma and presented as mean ± SE. Fasted blood was collected in the morning after overnight fasting.

As we discussed, there was a tendency to a lesser weight gain in rapamycin-treated mice, albeit only transiently statistically significant (Fig. 1 and Fig. S1, Supporting information). Levels of leptin mirror body weight, consistent with leptin production by fat cells. Importantly, rapamycin treatment did not impair metabolic parameters: It did not increase triglyceride, insulin and IGF1 levels (Fig. 5). Glucose levels were similar in all groups including mice on LF diet. There was no indication on either insulin resistance or overt diabetes in rapamycin-treated groups. This conclusion is supported by comparison of fasted and nonfasted (fed) levels of glucose and insulin (Fig. S5, Supporting information). Glucose levels were tightly controlled and were not increased in nonfasted mice compared with fasted mice (Fig. S5, Supporting information). In agreement, insulin levels were induced in fed mice, controlling glucose levels. In some mice in rapamycin-treated R2 group, there was a strong induction of insulin in response to food, suggesting preserved beta-cell function. As a small number of surviving mice in control HF group precluded statistically significant differences, we used correlations between parameters to estimate potential changes. It was shown that rapamycin decreased body weight gains on HF diet (Chang *et al*., 2009). We found a strong correlation between weight and leptin levels (Fig. S6, Supporting information). Leptin levels also correlated with insulin, glucose and IGF-1 levels (Fig. S6, Supporting information)**.** Except for one mouse in control HF group, other HF control mice tended to have higher leptin, glucose, IGF-1 and insulin levels compared with mice in R2 group (Fig. S6, Supporting information).

## Discussion

Here, we demonstrated for the first time that rapamycin extended lifespan in obese mice on high-fat (HF) diet. A significant increase in survival was achieved by intermittent administration of rapamycin. The most prominent effect was observed in mice treated once a week. All mice in this (R2) group survived until the end of experiment. Two other schedules of rapamycin administration (every other week: R1 and R3) showed tendency to decrease mortality, albeit statistical significance was not achieved. Nevertheless, survival in all three rapamycin-treated groups combined (80%) was significantly higher (*P* < 0.05) than in control HF mice (40%). Noteworthy, this effect was achieved in male mice, which according to previous studies are less responsive to rapamycin compared with female mice (Harrison *et al*., 2009) (Miller *et al*., 2011; Leontieva *et al*., 2012a). It is remarkable that rapamycin was administrated only once a week. Weekly administration of rapamycin partially reduced obesity, but this cannot completely explain the improvement in overall survival. In fact, mortality in lean mice group (control mice on low-fat diet – LF group) was higher than in R2 mice, even though difference in survival was not statistically significant at the end of experiment (Fig. S7, Supporting information). Small group size is one of limitations of this study. Another limitation is slightly different prehistory between R1, R2 and R3 groups. Prior beginning of current study, mice in R3 and R1 groups received low dose of resveratrol for 3 months. However, we believe that this difference is not consequential for several reasons: (i) at the end of the pretreatments, three groups were similar in metabolic parameters; (ii) the group (R2) that showed the best result in the current study did not receive resveratrol, so we cannot attribute the survival effect of rapamycin in this group to prehistory of resveratrol administration.

We also evaluated metabolic parameters in survived mice. Rapamycin treatment was discontinued 8 days before mice were sacrificed, to eliminate direct inhibitory effects of rapamycin on mTOR/S6 pathway. Rapamycin-treated mice did not develop metabolic abnormalities by the end of the study. Fasting levels of glucose, triglyceride and insulin were normal. Leptin and IGF-1 levels showed the tendency to be lower in R2 group than in control HF group. Levels of p-Akt (S473) tended to be higher in R2 mice compared with control mice, indicating that intermittent rapamycin did not obviously affect mTOR complex two, which phosphorylates p-Akt at S473. Levels of phosphorylated S6 (p-S6), a marker of mTOR/S6K activity, were statistically significantly lower in both the cardiac muscle and the liver in R2 group, received rapamycin every week, than in control HF group. Intriguingly, as shown by Sengupta *et al*. in figure 4C, fasting levels of p-S6 are higher in old mice than in young mice (Sengupta *et al*., 2010a). Given that p-S6 levels distinguish quiescence from senescence (Blagosklonny, 2012a; Leontieva *et al*., 2012b), fasting levels of p-S6 may be a potential marker of aging. Levels of p-S6 in the heart muscle are correlated with body size and tend to be higher in male than in female mice (Leontieva *et al*., 2012a). This is in agreement with observations that big males tend to live shorter than females and small strains of mice. Taken together, these data support the notion that the mTOR/S6K pathway drives both growth and aging **(**Blagosklonny & Hall, 2009).

mTOR is activated by insulin, IGF-1 and inflammatory cytokines (Zoncu *et al*., 2011; Cornu *et al*., 2012), all of which are markers of fast aging and poor health. Furthermore, activated mTOR/S6K pathway can cause insulin resistance (Khamzina *et al*., 2005; Krebs *et al*., 2007). Therefore, lower fasting levels of p-S6 may be a marker of slower aging and metabolic health. Our study probably underestimated positive effects of rapamycin, because metabolic parameters were evaluated only in mice that survived until the age of 2 years. Whereas all mice in R2 group survived until the end of experiment, only 40% mice survived in control group. In control HF group, mice with shorter lifespan (less healthy and faster aging by definition) did not survive and thus were not included in the investigation of p-S6 and metabolic parameters.

This study is a starting point to further evaluate intermittent schedules of rapamycin and to increase their life-extending potential by modulating doses and frequency. The life extension by intermittent treatment with rapamycin may be further potentiated with diet, the antidiabetic drug metformin (Anisimov *et al*., 2011a; Martin-Montalvo *et al*., 2013) and, if possible, physical exercise. Taken together, these modalities may improve health and increase lifespan in aging humans.

## Experimental procedures

### Mice

All animal studies were conducted in accordance with the regulations of the Committee of Animal Care and Use at Roswell Park Cancer Institute.

Prehistory [previous study (Leontieva *et al*., 2013c)]: 9-month-old male mice (C57BL/6NCr strain) were divided into 5 groups: one group received standard laboratory chow (5% fat, low fat) (LF) diet. Four other groups received high-fat 60% diet (Research Diets, Inc, Cat # D12492 Rodent Diet 60% kCal% fat; New Brunswick, NJ, USA) (HF) for 3 months. These four groups on HF diet were as follows: HF group – control (HF), R1 group received orally low dose of resveratrol; R2 group received orally low dose of rapamune; and R3 group received a combination of resveratrol and rapamune, as described previously (Leontieva *et al*., 2013c).

### Current study

One week after the first study ended, experimental groups (R1–R3) were treated with rapamycin (LC Laboratories) via i.p injections, according to the following schedules: R1 group received 1.5 mg kg^−1^ three times/week/every other week; R2 group was injected with 1.5 mg kg^−1^ week^−1^ per every week; and R3 group was administered 0.5 mg kg^−1^ three times/week/every other week. Treatment was continued for 11 months, and weight was measured every week. On the eighth day after the last treatment, mice were fasted overnight and sacrificed. Blood was collected at the end of the day before food was removed for overnight fasting. Next morning, fasted blood was collected and mice were sacrificed. Nonfasted and fasted plasma were prepared, accordingly, for biochemical analysis.

Rapamycin (LC Laboratories, Woburn, MA, USA) was dissolved in ethanol at 15 mg mL^−1^ (stock) and then diluted to 0.15 mg mL^−1^ in PBS containing 5% Tween-80, 5% PEG 400 and 4% ethanol.

### Immunoblot analysis

Tissues were homogenized, and immunoblotting was performed as previously described (Leontieva *et al*., 2012a). Rabbit antiphospho S6(Ser 240/244), antiphospho-AKT(Ser473), total AKT, and anti-S6 were used by us as previously described (Leontieva *et al*., 2012c) and purchased from Cell Signaling Biotechnology (Danvers, MA, USA); monoclonal anti-β-actin –peroxidase (AC-15) and mouse anti-GAPDH antibodies were obtained from Sigma-Aldrich (St. Louis, MO, USA) and Invitrogen (Grand Island, NY, USA), respectively.

Glucose levels in blood plasma were measured using Accu-Chek Aviva strips (McKesson, Atlanta, GA, USA).

Insulin, IGF1, leptin, and triglyceride concentration in blood plasma, were measured using Insulin (Mouse) Ultrasensitive ELISA kit (ALPCO Diagnostics, Salem, NH, USA), IGF1 (Mouse/Rat) ELISA kit (ALPCO), Mouse Leptin ELISA kit (Crystal Chem Inc, Downers Grove, IL, USA), and Triglyceride Colorimetric Assay kit (Cayman Chemical Company, Ann Arbor, MI, USA), respectively. Data were analyzed using range of standards and four parameter logistic fit or linear regression.

### Statistical analysis

*T* test and correlation analyses [Pearson *r* coefficient and *P* value (two tailed)] were performed using GraphPad Prism version 5.00 for Windows, GraphPad Software, San-Diego California, USA. www.graphpad.com.

## Supporting Information

**Fig. S1** Percent of initial body weight.

**Fig. S2** Kaplan–Meier survival curves for control mice on HF diet (HF, n = 10) and mice treated with schedule R1 (n = 10) and R3 (n = 9).

**Fig. S3** Commassie Blue-stained membrane of liver blots shown in Figure 4A to confirm equal loading.

**Fig. S4** Levels of p-S6 and p-AKT in kidneys of surviving mice.

**Fig. S5** Levels of fasted and nonfasted glucose and insulin in blood plasma of male mice.

**Fig. S6** Correlation between metabolic parameters in fasted plasma.

**Fig. S7** Kaplan–Meier survival curves.

**Table S1** Survival curve high-fat control (HF) and high fat + rapamycin-treated group 2 (HF+rapamycin (group 2)).

**Table S2** Survival curve high-fat control (HF) and high fat + rapamycin-treated groups (HF+rapamycin all groups).
