# Supplementary figures and images for: Weekly administration of rapamycin improves survival and biomarkers in obese male mice on high-fat diet
Source: Aging Cell. 2014 Mar 22;13(4):616–22. doi: 10.1111/acel.12211 (PMC4326934; doi:10.1111/acel.12211)

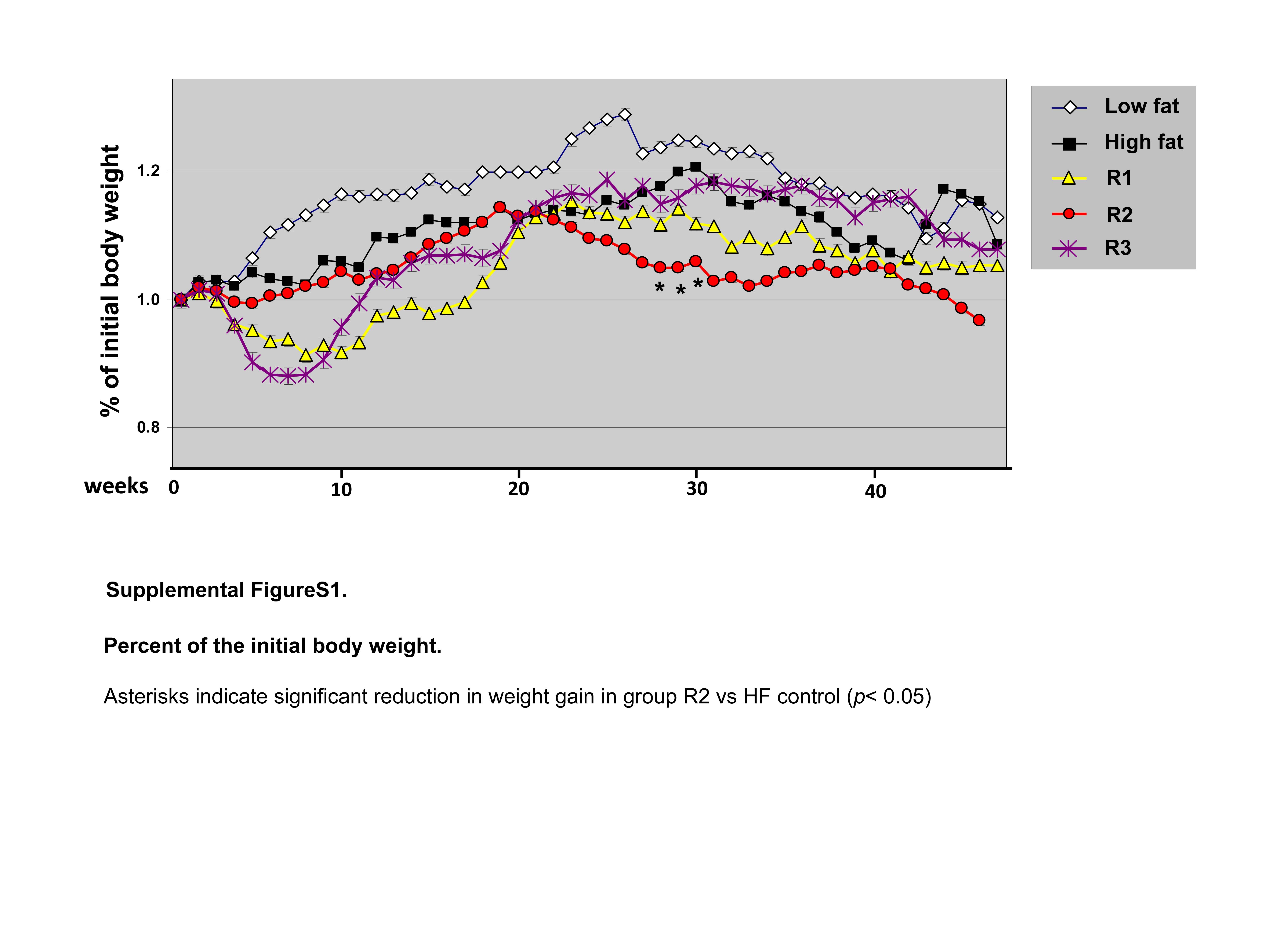

Supplement: Supplementary file 1 — Fig. S1 Percent of initial body weight. [file acel0013-0616-sd1.jpg]

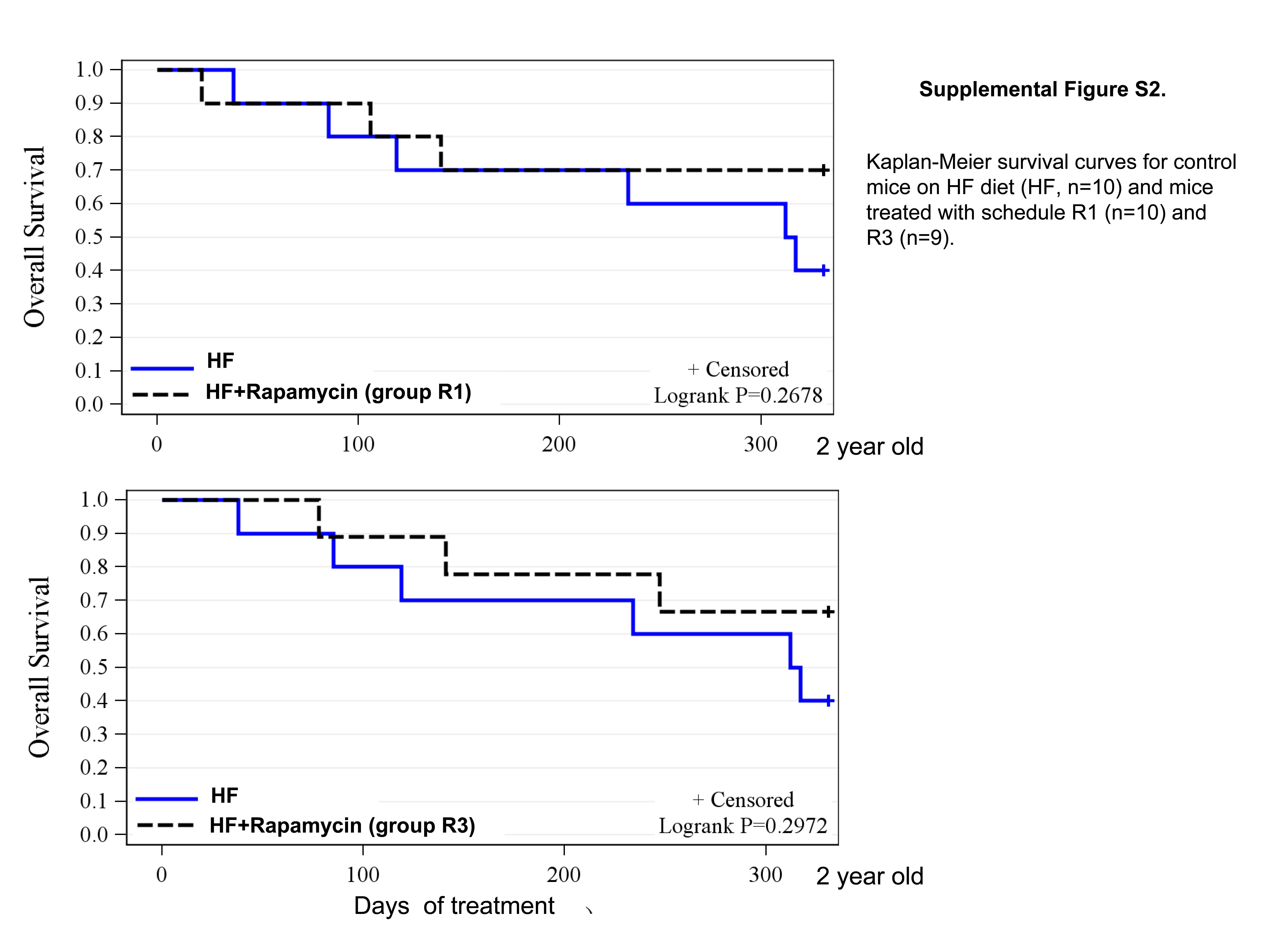

Supplement: Supplementary file 2 — Fig. S2 Kaplan–Meier survival curves for control mice on HF diet (HF, n = 10) and mice treated with schedule R1 (n = 10) and R3 (n = 9). [file acel0013-0616-sd2.jpg]

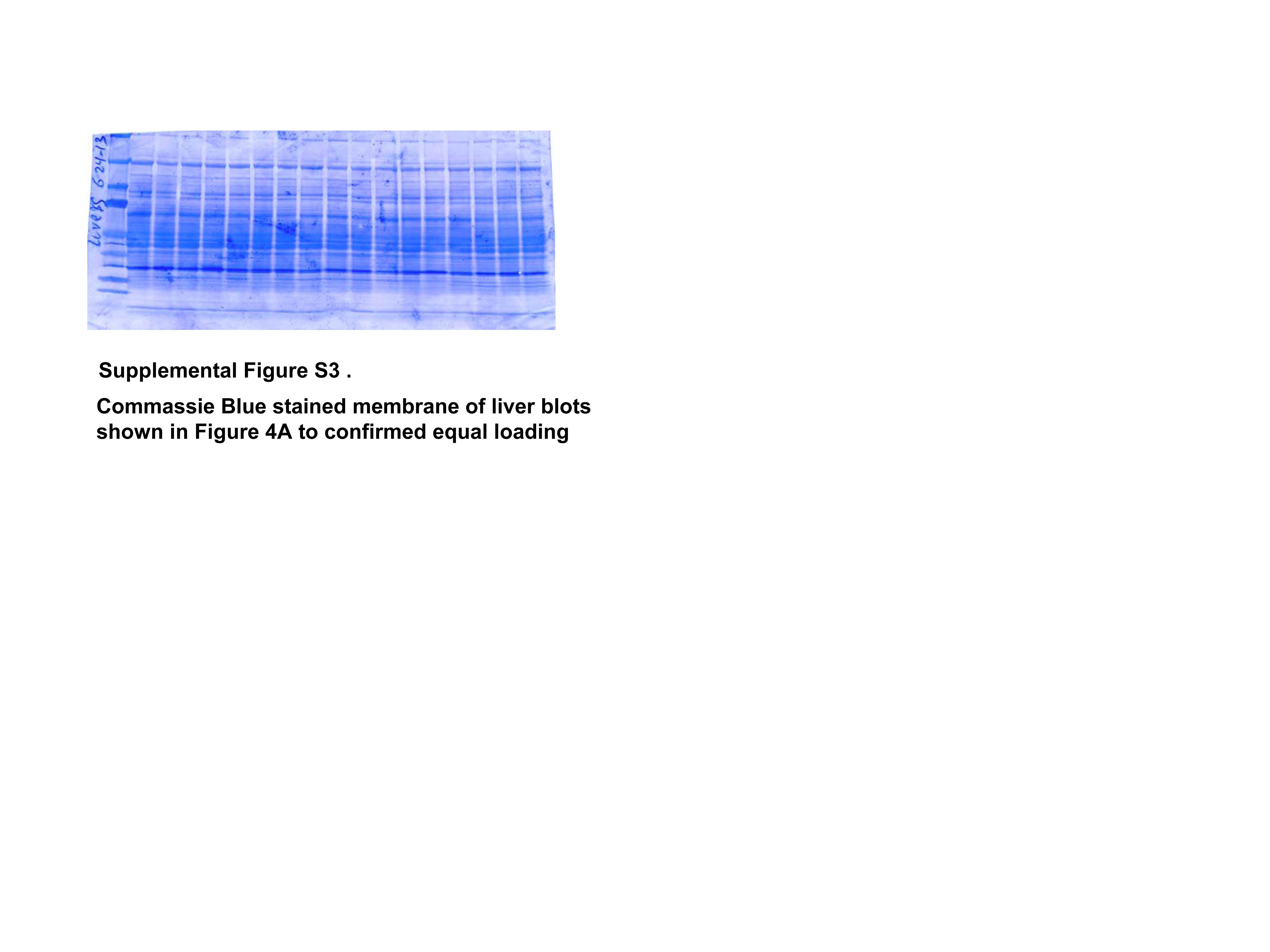

Supplement: Supplementary file 3 — Fig. S3 Commassie Blue-stained membrane of liver blots shown in Figure 4A to confirm equal loading. [file acel0013-0616-sd3.jpg]

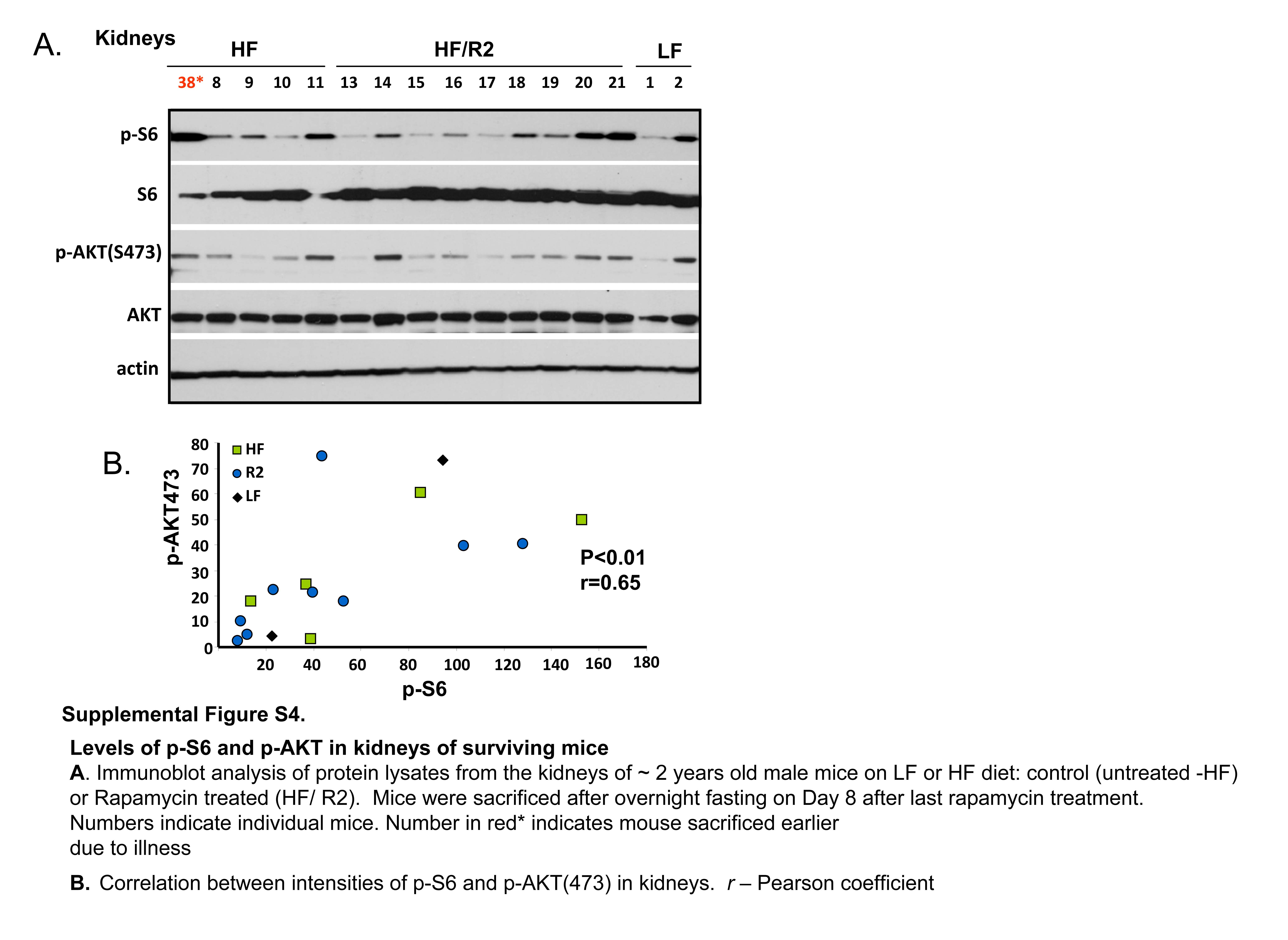

Supplement: Supplementary file 4 — Fig. S4 Levels of p-S6 and p-AKT in kidneys of surviving mice. [file acel0013-0616-sd4.jpg]

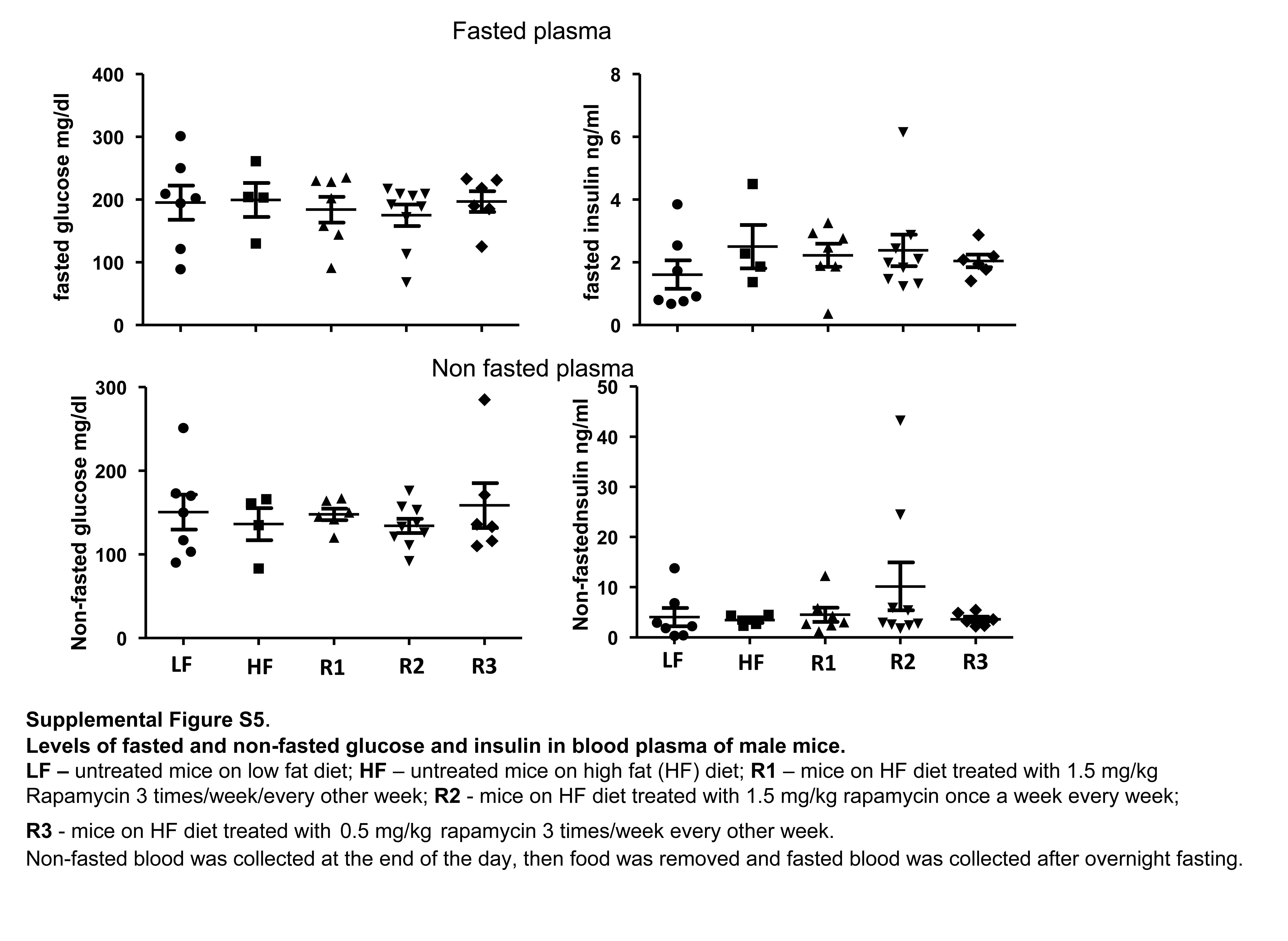

Supplement: Supplementary file 5 — Fig. S5 Levels of fasted and nonfasted glucose and insulin in blood plasma of male mice. [file acel0013-0616-sd5.jpg]

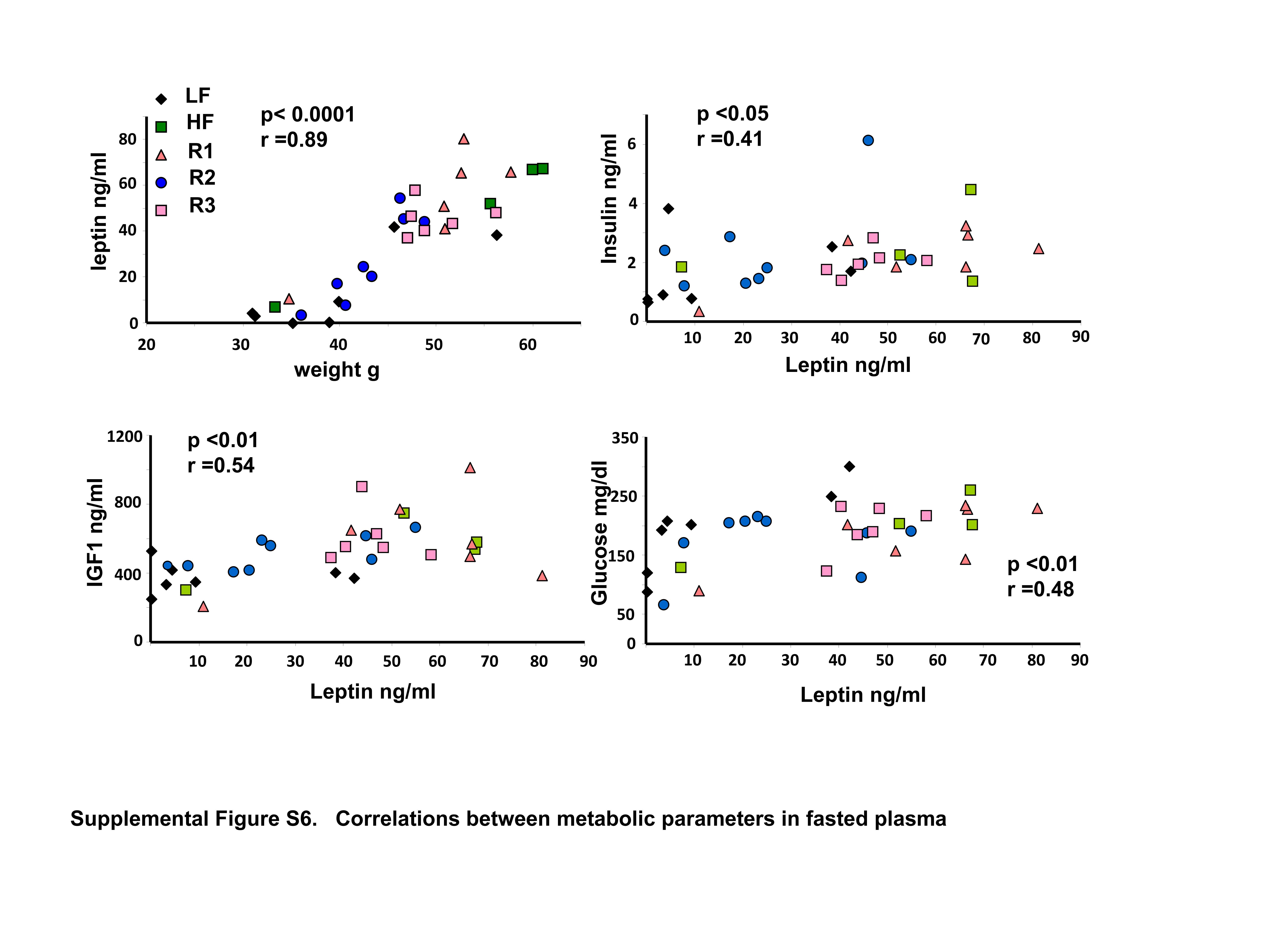

Supplement: Supplementary file 6 — Fig. S6 Correlation between metabolic parameters in fasted plasma. [file acel0013-0616-sd6.jpg]

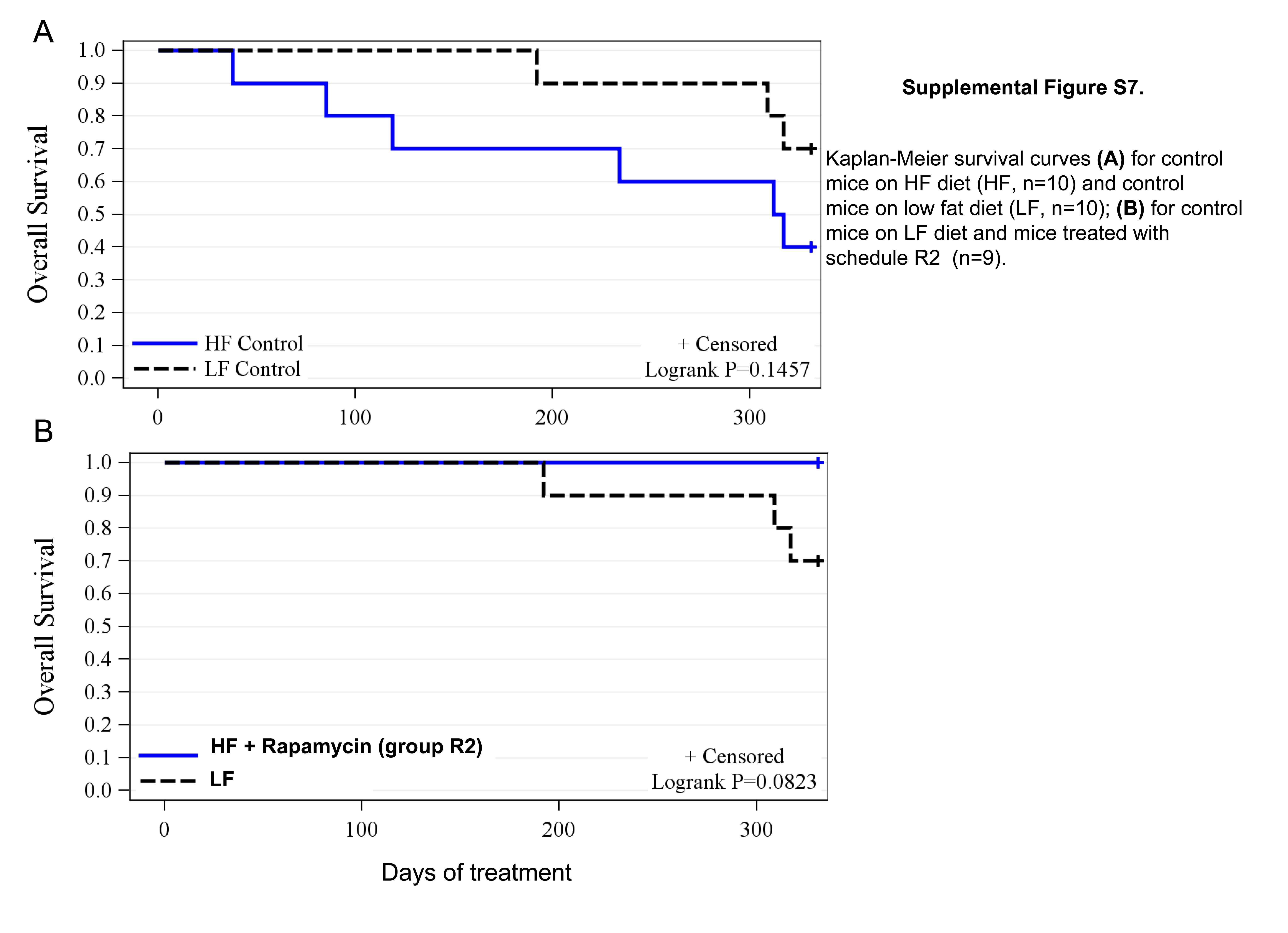

Supplement: Supplementary file 7 — Fig. S7 Kaplan–Meier survival curves. [file acel0013-0616-sd7.jpg]
